# Morphologically Switchable Twin Photonic Hooks

**DOI:** 10.3390/ma17194695

**Published:** 2024-09-24

**Authors:** Zejie Shi, Kaihua Wei, Pinghui Wu, Bohuan Chen, Shanhui Fan

**Affiliations:** 1 Key Laboratory of Micro-Nano Sensing and IoT of Wenzhou, Wenzhou Institute of Hangzhou Dianzi University, Wenzhou 325038, China; 232060255@hdu.edu.cn (Z.S.); 212060228@hdu.edu.cn (B.C.); 2 Fujian Provincial Key Laboratory for Advanced Micro-Nano Photonics Technology and Devices & Key Laboratory of Information Functional Material for Fujian Higher Education, Quanzhou Normal University, Quanzhou 362000, China; phwu@qztc.edu.cn

**Keywords:** twin photonic hooks, FDTD, bending angle, full width at half maximum (FWHM)

## Abstract

A dual fan-shaped structure covered with Ag films was investigated for generating twin photonic hooks (t-PHs). The t-PH characteristics of this structure are studied using the Finite-Difference Time-Domain (FDTD) method. The results show that by designing appropriate fan-shaped opening angles and angles of Ag films coverage, the switching between t-PHs, S-shaped t-PHs, and W-shaped t-PHs can be achieved, along with controlling over the bending angles. The maximum first, second, and third bending angles for the obtained W-shaped t-PHs are 51.3°, 36.4°, and 41.8°, respectively, while the Ag films angle is 5°. The investigated tunable morphology t-PHs provide innovative applications in the fields of nanolithography and integrated optics.

## 1. Introduction

Photonic nanoscale jets (PNJs) are an advanced optical phenomenon which have important scientific and application potential. Due to their excellent characteristics such as high spatial resolution, high light intensity, significant local gain, controllable direction, and adjustable intensity, photonic nanoscale jets (PNJs) have been widely applied in fields such as optical storage [1], micro–nano-manipulation [2], super-resolution imaging [3], biomedical imaging, and environmental monitoring. In order to further expand the application range of PNJ, I.V. Minin and O.V. Minin introduced a new kind of curved light beams called the photonic hook (PH) in 2015, which realized the bending based on PNJs [4]. This concept is based on the properties and behavior of light beams and opens up new directions in optical research. In addition, this discovery greatly promoted the research and development of PNJs-related fields and stimulated the emergence of acoustic hooks [5] and plasma hooks [6]. Various research methods have been proposed to adjust PH parameters such as length, curvature, and full width at half maximum (FWHM) of the PH [4,7,8,9,10,11,12]. In addition, in 2023, I.V. Minin and O.V. Minin proposed and demonstrated a new type of time-domain self-bending photonic hook (time-PH) generated through freezing water droplets, opening up new avenues in temporal optics [13]. In the same year, Zhang et al. studied the PH generated by the interaction between a rotating dielectric sphere and a plane wave with a certain angular velocity [14]. Scholars have also studied multi-segmented bent photonic hooks. In 2022, Yu et al. designed a tunable optical structure and discovered switchable W-shaped and J-shaped photonic hooks for the first time by controlling the propagation path and mode of light [15]. This discovery expanded the range of applications of photonic hooks, giving them more potential in optical imaging, communication, and micromanipulation. Xu et al. generated an S-shaped photonic hook using microspheres covered with a silver film, which demonstrated a longer effective propagation length than traditional photonic hooks [16]. This provides a new method for particle trapping by bypassing multiple obstacles. This innovative photonic hook not only advances fundamental research in photonics, but also has a wide range of potential applications, such as in advanced imaging techniques and optical devices.

The above research mainly focus on the generation of individual PH, but there are still some shortcomings in the field of multi-objective operation. It was not until the emergence of twin photonic hooks (t-PHs) that the gap in this field was filled. In 2017, Poteet et al. designed two inclined beams to irradiate microspheres and successfully generated t-PHs by using the finite-difference time-domain (FDTD) method [17]. The finite-difference time-domain (FDTD) method is a numerical technique used to solve electromagnetic field problems. The advantage of this method lies in its intuitiveness and wide application range. Although its calculation cost is large, it can deal with complex geometric shapes and a variety of materials, which makes it suitable for solving complex electromagnetic problems. Subsequently, Poteet and colleagues confirmed the results of this theoretical study in further experiments in 2018 [18]. In 2020, Zhou used two adjacent parallel cylinders to generate t-PHs under TE-polarized plane wave irradiation [19]. In 2023, Yang et al. designed a multi-medium structure composed of periodically arranged scattering units to generate t-PHs using the FDTD method [20]. In addition, previous studies had deeply discussed the technology of using a fan-shaped structure to generate t-PHs [21]. Unfortunately, although our predecessors have made remarkable progress in this field, there is currently no work that can achieve the shape switching of twin photonic hooks and enable multi-segment bending.

In view of the above research status and the problems in related fields, a dual fan-shaped structure is proposed in this work and the optical focusing characteristics under plane wave illumination are investigated. By changing the opening angle of the fans and the coverage angle of Ag films, the morphological switching of photonic hooks between W-shaped t-PHs, S-shaped t-PHs, and t-PHs, as well as control over the bending angle, can be achieved. The research findings have potential applications in super-resolution imaging, micro-machining, optical trapping, sensor technology, and other fields.

## 2. Materials and Methods

**Figure 1:**
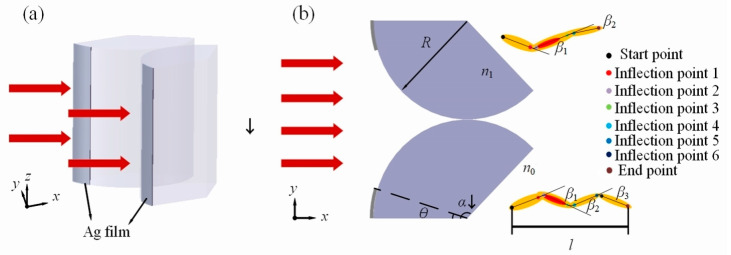
Schematic diagram of a dual fan-shaped cylinder illuminated by a plane wave: (**a**) 3D stereogram; (**b**) 2D cross-section diagram. Red arrows represent plane waves.

The model diagram is shown in Figure 1. It shows an infinite dielectric fan-shaped structure with silver films, irradiated by a plane wave with a wavelength of λ = 632 nm. The dual fan-shaped structures are symmetrically distributed along the x = 0 axis, forming t-PHs in the shadow region. Given the finite-difference time-domain (FDTD) method’s effectiveness in accurately describing the propagation of light waves in media [22], the FDTD algorithm-based solution method is adopted to obtain the optical field on the shadow side of the dual fan-shaped cylinder. The radius of the fan-shaped structure is R = 5 μm, the opening angle of the fan-shaped structure is α, the coverage angle of the silver films is θ, and the thickness of Ag films is 50 nm. Since BaTiO3 with a refractive index of 1.90 is a commonly used high refractive index dielectric material in microsphere-based applications [23,24] and capacitor materials [25], the refractive index of the fan-shaped structure is set as n_1_ = 1.90. The background refractive index n_0_ is set to 1, the same as that of air.

In this model, a non-uniform grid is employed throughout the entire computational domain, with all grid elements sized at λ/12. Perfectly matched layers (PML) are implemented as field boundaries to eliminate any reflection effects. The light intensity distribution exhibits a curvilinear structure, which gradually becomes straight after a certain distance. The S-shaped t-PHs structure can be defined by its starting point (SP), four inflection points (IP1, IP2, IP3, and IP4), and endpoint (EP) [16]. The W-shaped t-PHs structure can be defined by its starting point (SP), six inflection points (IP1, IP2, IP3, IP4, IP5, and IP6), and endpoint (EP). These points are located by slicing the t-PHs and identifying the position of maximum light intensity. After fitting the points of maximum light intensity, a smooth curve, referred to as the centerline, is obtained, and the length of the centerline represents the propagation length l of the t-PHs.

The inflection points are the locations where the curvature of the centerline changes in the t-PHs structure [15]. The endpoint of this study is defined as the point on the centerline of the t-PHs with an intensity enhancement factor of Imax/e [11,26]. Imax represents the maximum enhancement of |E|2 that occurs on the shadow side. It quantifies the enhancement factor of |E|2 compared to the incident light. Based on these points, the curvature of the S-shaped t-PHs can be defined by the bending angles β1 and β2; as shown in Figure 1, β1 is the angle between the lines connecting SP to IP1 and IP2 to IP3, respectively. β2 is the angle between the lines connecting IP2 to IP3 and IP4 to EP, respectively. The W-shaped t-PHs can be defined by the bending angles β1, β2 and β3, β1 is the same as that in the S-shaped t-PHs; β2 is the angle between the lines connecting IP2 to IP3 and IP4 to IP5, respectively, and β3 is the angle between the lines connecting IP4 to IP5 and IP6 to EP, respectively.

## 3. Results and Discussion

### 3.1. Comparison with and without Ag Films

**Figure 2:**
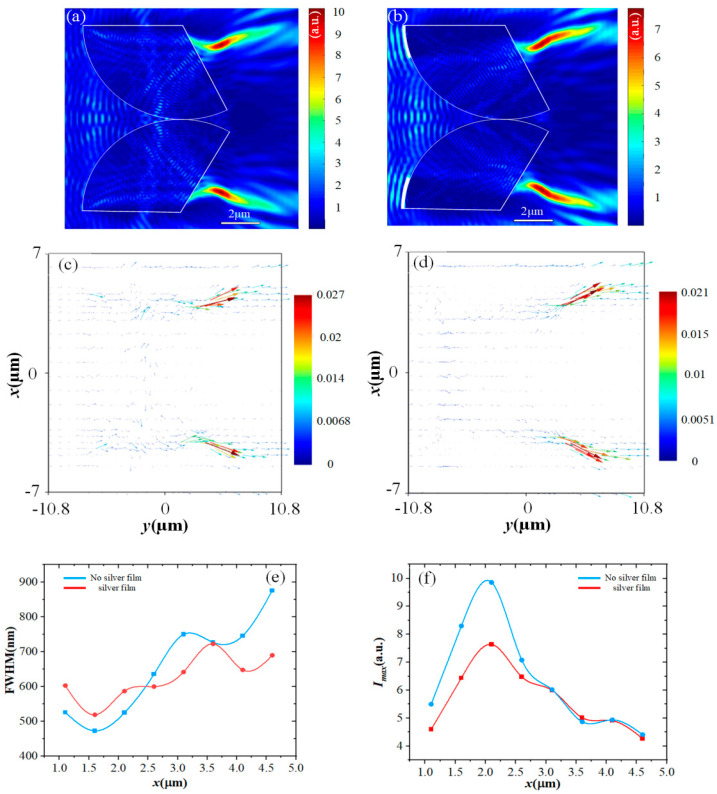
(**a**) Light field produced by the dual fan-shaped cylinder without Ag films coverage. (**b**) Light field produced by the dual fan-shaped cylinder with Ag films coverage. (**c**) Poynting vector without Ag films. (**d**) Poynting vector with Ag films. (**e**) The change in FWHM along the x-axis. (**f**) Variation of Imax along the x-axis.

The differences between t-PHs generated with or without Ag films covering the dual fan-shaped cylinder were compared. The opening angle α of the sector was set to 120° and the covering angle θ of Ag films was set to 20°. The spatial distribution of the optical field is shown in Figure 2a,b. It can be observed that due to the disruption of the optical symmetry by the upper and lower sectorial elements, an asymmetric spatial distribution of the energy flow occurs inside the sectoral cylinder, leading to the generation of t-PHs without the presence of a silver film. The existence of the Ag thin film leads to a pronounced secondary bending of the t-PHs, resulting in a more uniform internal optical field intensity distribution and the formation of S-shaped t-PHs. As shown in Figure 2c,d, comparing the Poynting vector of the optical field before and after the bi-sectoral cylinders are covered with the Ag film, it can be observed that the application of the Ag film reduces the energy flow inside the sectoral cylinders. The incident light excites surface plasmon polaritons (SPP) on the surface of the Ag film, significantly altering their propagation direction. SSP is a coupling mode of collective vibration of free electrons on the surface of light and metal, which exists at the interface of metal and medium and has the characteristics of light and metal electron. The superposition of SPP waves and conventional PH waves leads to the formation of this S-shaped intensity distribution. SPP waves are EM modes propagating along the metal–dielectric interface, where the surface collective excitation of free electrons in the metal is coupled to the transitory EM field in the dielectric layer. It has been reported that metal thin films can serve as SPP waveguides for transmitting EM energy [27]. Additionally, Minin et al. theoretically and experimentally demonstrated the generation of plasmonic hooks based on SPP excitation [28,29].

To demonstrate the properties of the light field, the t-PHs were sliced along the x-axis and the full width at half maximum (FWHM) of the intensity distribution was recorded. In Figure 2e, it was observed that at a propagation distance of 4.7λ, the S-shaped t-PHs maintained an FWHM of 1λ, while the conventional t-PHs had a FWHM of 1.2λ. Furthermore, the fluctuation of the S-shaped FWHM is smaller compared to conventional t-PHs. The position and value of the maximum intensity in each slice were also recorded, as depicted in Figure 2f. At a propagation distance of 5.7λ, the intensity of the S-shaped t-PHs decreases to 56% of the maximum value, while the intensity of conventional t-PHs decreases to 44% at a propagation distance of 5.7λ. The decrease rate of the S-shaped t-PHs is smaller, and they also have a longer effective length.

### 3.2. Influence of Difference Opening Angles

**Figure 3:**
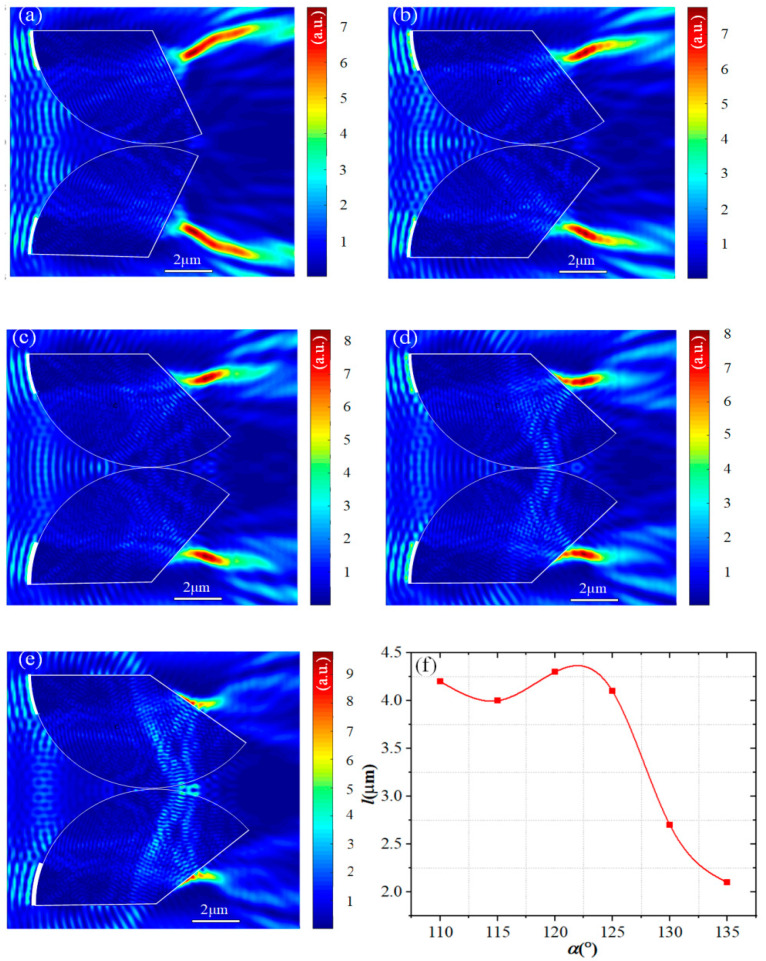
(**a**–**e**) Light fields generated by different opening angles α. (**a**) α = 110°. (**b**) α = 115°. (**c**) α = 125°. (**d**) α = 130°. (**e**) α = 135°. (**f**) The propagation length l of t-PHs is generated by different opening angles α.

Subsequently, the influence of different opening angles *α* on the light field was studied. While keeping the Ag films coverage angle at 20°, the opening angle *α* varied from 110° to 135°. When *α* is 110°, a traditional t-PHs is formed (Figure 3a). Increasing the opening angle *α* to 115–120° (Figure 3b), a secondary bending is formed in the middle section of the t-PHs, resulting in an S-shaped t-PHs. Continuing to increase the opening angle α to 125° (Figure 3c), the t-PHs tail beams begin to bend upwards and downwards, forming a W-shaped t-PHs. As the opening angle α continues to increase to 130° (Figure 3d), the t-PHs tail starts to become faint and the intensity significantly weakens. When the opening angle *α* is 135° (Figure 3e), the t-PHs almost overlap with the wall of the fan-shaped cylinder, and the bending characteristics of the tail beams completely disappear. Based on the SPP electric field formula

(1) $$E(z>0)=(E_{x}^{0},\;0,\;E_{z}^{d})\cdot e^{i(k_{SPP}x-\omega t)}\exp(-\sqrt{k_{SPP}^{2}-\varepsilon_{d}k_{0}^{2}})$$

(2) $$E(z<0)=(E_{x}^{0},\;0,\;E_{z}^{m})\cdot e^{i(k_{SPP}x-\omega t)}\exp(-z\sqrt{k_{SPP}^{2}-\varepsilon_{m}k_{0}^{2}})$$

where $E_{x}^{0}$ and $E_{z}^{d(m)}$ are the amplitudes of the corresponding electric-field components in the dielectric (metal) medium, $\varepsilon_{d}$ and $\varepsilon_{m}$ are the permittivity’s of the dielectric and metal, respectively, $k_{0}$ is the light wavenumber ( $k_{0}=2\pi /\lambda$, λ is the light wavelength) and $k_{SPP}=k_{x}$ is the mode propagation (complex) constant yet to be determined. It can be deduced that the electric field of SPP waves decays exponentially into two adjacent media. Therefore, as the opening angle increases, the electric field exhibits an exponential decrease, gradually weakening the impact on the tail of t-PHs and resulting in the disappearance of bending characteristics. From Figure 3a–e, it can also be observed that the enhancement of *I*_max_ relative to the incident light increases with the opening angle *α*. The enlarged scattering elements increase the optical path of the light passing through them, resulting in a more compact focal spot. The morphology and focal intensity depend on the different opening angles *α*.

The propagation length l of t-PHs generated by different opening angles α was also measured, as illustrated in Figure 3f. It can be observed that within the range of opening angles from 110° to 115°, the transmission length l decreases from 4.1 μm to a value of 4.0 μm. Within the subsequent range from 115° to 122°, the transmission length l then increases, reaching a maximum value of 4.4 μm. After the opening angle α exceeds 125°, the propagation length l steadily decreases. This is because the increase in opening angle brings the fan-shaped boundaries closer to the SP, hindering the generation and bending of the tail beams. Furthermore, when the total energy of the dual dielectric structure system remains constant; the higher the peak intensity (Imax) of the PH, the more concentrated the energy, and thus the shorter the propagation length. Conversely, when the energy is more dispersed, the propagation length becomes longer.

### 3.3. The Influence of Ag Films Coverage Angle

**Figure 4:**
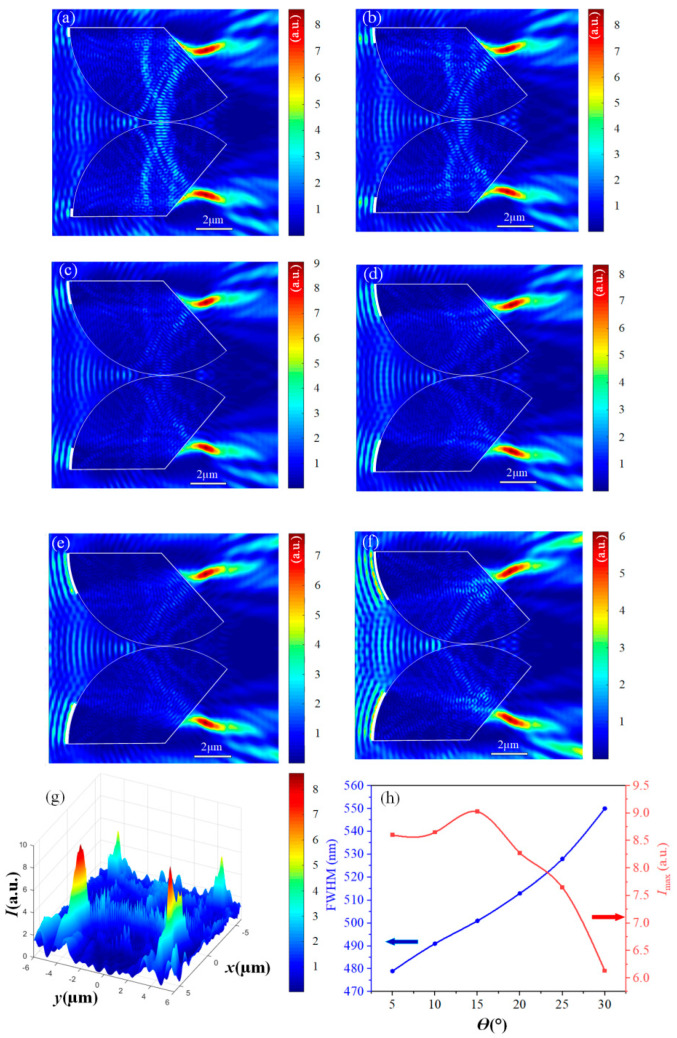
(**a**–**e**) Light fields generated by different Ag film-covering angles θ. (**a**) θ = 5°, (**b**) θ = 10°, (**c**) θ = 15°, (**d**) θ = 20°, (**e**) θ = 25°, (**f**) θ = 30°. (**g**) 3D light field image at a silver film angle of 10°. (**h**) Imax (red line) and FWHM (blue line) at different Ag film-covering angles θ.

Next, the coverage angle θ of Ag films was varied to study its influence on t-PHs. Keeping the opening angle α at 130°, the coverage angle θ of the Ag films varied from 5° to 30°. During the variation of θ from 5° to 20° (Figure 4a–d), the combined effects of SPP waves and PH waves lead to the formation of a W-shaped t-PHs on the shaded side of the fan shape. This phenomenon can be attributed to the gradual enhancement of the SPP wave effects as the SPP waves undergo light absorption and reflection on the metal surface, altering the distribution of the optical field and subsequently affecting the propagation of PH waves. As θ increases, the influence of SPP waves progressively strengthens, resulting in a reduction in the curvature of the bent tail beams on both sides. To provide a more intuitive visualization of the W-shaped t-PHs, a three-dimensional optical field image was generated at a silver film angle of 10° (Figure 4g). When θ further increases to 25–30° (Figure 4e,f), the formation of the tail beams is predominantly governed by SPP waves, with less influence from PH waves. Consequently, the bending characteristics of the t-PH tail almost disappear, giving rise to an S-shaped t-PH. The coverage angle θ of Ag films significantly impacts the morphological changes in t-PHs.

Further exploration was conducted on the influence of the coverage angle θ of the silver film on the maximum intensity (Imax) and the full width at half maximum (FWHM) of t-PHs. The line chart in Figure 4h illustrates the relationship between θ and the t-PHs characteristics. At θ = 5°, the FWHM is minimized, indicating a narrow FWHM distribution and strong focusing capability. This is attributed to the limited confinement of the light field within the Ag film at smaller θ angles, resulting in a more pronounced enhancement effect. With the increase in θ, the FWHM also increases approximately linearly. This trend suggests that the larger the coverage angle of the Ag film, the greater the partial impact on light focusing, leading to a broadening of the light intensity distribution. Between θ = 5° and θ = 15°, Imax exhibits a slight increase relative to the incident light. However, beyond this range, the energy entering the fan-shaped structure significantly decreases due to the increase in surface reflection of the Ag film. This leads to a significant decrease in Imax relative to the incident light, with a further increase in θ. In summary, the variation of the coverage angle θ of the Ag film not only affects the morphology of t-PHs, but also has profound implications for its focusing capability and intensity enhancement.

By changing the coverage angle θ of the Ag film, the curvature of the light intensity distribution can be effectively controlled. As shown in Table 1, the first bending angle (β1), the second bending angle (β2), and the third bending angle (β3) of the generated light field exhibit clear trends in response to the variation of the coverage angle θ of the Ag film. Specifically, β1 decreases with an increase in coverage angle θ, indicating that as the coverage of the Ag film becomes wider, the curvature of the light intensity distribution towards the center becomes less pronounced, with this effect reaching a maximum value of 51.3° at θ = 5°. β2 decreases with the increase in the coverage angle θ of the Ag film when forming the w-type t-PHs, reaching a maximum of 36.4° at θ = 5°. The increased coverage angle of the Ag film inhibits the formation of β3 angle. As the coverage angle θ increases, β3 decreases, indicating that the curvature of the t-PHs in the region decreases as the coverage range of the silver film expands. At θ = 5°, the maximum value of β3 reaches 41.8°, indicating that at this angle, the inhibitory effect is minimal and the curvature of the light intensity distribution is more pronounced. In summary, controlling the coverage angle θ of the Ag film is an effective means of adjusting the curvature of the light intensity distribution. The observed variations of β1, β2, and β3 angles with θ can be customized to the required light field configuration for different applications.

## 4. Conclusions

This paper proposed and studied the method of generating t-PHs using a dual fan-shaped structure covered with Ag films. By designing appropriate fan-shaped opening angles and angles of Ag films coverage, the switching between t-PHs, S-shaped t-PHs, and W-shaped t-PHs can be achieved, along with control over the bending angles. The maximum first, second, and third bending angles of the W-shaped t-PHs are 51.3°, 36.4°, and 41.8°, respectively, when the Ag films angle is 5°. This study provides a new method for preparing t-PHs using dielectric particles, with potential applications in nanolithography and electromagnetic wave manipulation. In addition, this method may be of great help to the research fields of light sensors, optoelectronic devices, and biomedicine in the future.

## Figures and Tables

**Table 1:** Bending angle β of t-PHs under different Ag film-covering angles θ.

Angle of Silver Films	*β*_1_	*β*_2_	*β*_3_
5°	51.3°	36.4°	41.8°
10°	50.5°	35.3°	41.6°
15°	48.2°	21.1°	13.6°
20°	47.6°	13.3°	12.8°
25°	34.1°	19.5°	/
30°	33.6°	29.2°	/

## Data Availability

Data will be made available on request.
